# Predicting “When” Using the Motor System’s Beta-Band Oscillations

**DOI:** 10.3389/fnhum.2012.00225

**Published:** 2012-08-02

**Authors:** Luc H. Arnal

**Affiliations:** ^1^Department of Psychology, New York UniversityNew York, NY, USA

Anticipating future sensory events is one keystone of adaptive behavior. This notion is at the origin of recent theories suggesting perception and action control rely on internal models that are constantly tested and updated as a function of incoming sensory inputs. These hierarchical models (predictive coding and other generative models based on the notion of inference) suggest that neural responses reflect the difference between top-down expectations (or priors) and incoming feed-forward sensory inputs (Rao and Ballard, 1999; Friston, 2005).

Priors are formed via the extraction of statistical regularities and can therefore relate to many different dimensions of a sensory event (e.g., spatial, spectral, temporal…; Arnal and Giraud, 2012). In the temporal domain, isochronous rhythm arguably constitutes the most basic regularity that can be used to anticipate the occurrence of an event. Consistent with such a notion of predictive timing, temporally anticipating a sound in an isochronous stream reduces the uncertainty about its occurrence (Rohenkohl et al., 2012) and therefore leads to a decrease of the amplitude of electrophysiological responses in the auditory cortex (Costa-Faidella et al., 2011). The neural origin and computations supporting predictive timing remain unclear, but a new study by Fujioka et al. (2012) raises *a potential function of sensorimotor beta*-band *oscillations in the control of temporal anticipation* during beat perception.

The authors recorded neuromagnetic activity in participants that passively listened to isochronous (2.5, 1.7, and 1.3 Hz) or anisochronous (randomly spaced) tone sequences presented in four different blocks. Importantly, subjects’ attention was engaged in viewing a silent movie, and they were explicitly asked to ignore the sounds. Using a dipole source model of auditory responses, the authors first focused on the time-frequency profiles in response to these sequences in the auditory cortex. Consistent with previous findings, they observed transient increases of low (<10 Hz) and high frequency (>30 Hz) power, time-locked to the stimulus. More interestingly, while beta-band activity (13–25 Hz) consistently decreased after stimulus onset in every condition, the following beta rebound (*beta resynchronization*) increased differentially as a function of beat patterns. By evaluating the rising slope of the beta rebound for each condition, they found that beta rebound is fast and transient during aperiodic stimulation whereas it increased progressively to reach a maximum at the occurrence of the subsequent sound in the isochronous conditions. They also observed that the magnitude of the post-stimulus beta decrease co-varies with the frequency of stimulation (i.e., the slower the rhythm, the larger the beta decrease). Based on these findings, the authors suggest that beta rebound tracks the tempo of stimulation in the auditory cortex and can be used to maintain predictive timing.

Because beta-band activity is classically considered as being related to motor functions (Engel and Fries, 2010), the authors extended their investigation to whole-brain beta activity using a spatio-temporal principal component analysis. In addition to auditory regions, they identified a large network of sensorimotor regions implicated in the tracking of beat tempo. This suggests that the motor system is recruited during the passive perception of rhythms, even in the absence of any intention to move in synchrony with the beat. One might interpret this result as the passive, rhythmic entrainment of auditory, and motor systems. In that case, though, it would be unlikely to observe tempo-dependent beta rebounds following post-stimulus beta suppression. The fact that the beta rebound progressively increases and is maximal at the onset of upcoming sounds more likely supports an active, predictive timing account. These results suggest that (i) the motor system is automatically recruited during passive listening to anticipate forthcoming sounds, (ii) predictive timing allows to control neural activity in sensory regions, and (iii) beta-band oscillations play an instrumental role in predictive timing.

By examining in more detail the interactions between auditory and motor systems, the authors determined that post-stimulus event-related beta-coherence varied in an opposite way between these two systems. While beta-coherence decreased after stimulus onset in auditory regions, it simultaneously increased in motor areas. This may suggest that beta oscillations are used to control predictive timing via sensorimotor loops between auditory and motor systems. This interpretation must be viewed with caution in light of the absence of a clear causal relationship between the time courses of beta activity in these regions. However, these results converge to support a functional role of beta activity in the predictive modulation of auditory activity by the motor system.

## Oscillatory Processes in Predictive Timing

### Beta oscillations reflect top-down modulation of auditory activity

Current views suggest that oscillations reflect cyclic variations of neuronal excitability states and that their predictive modulation may participate to constrain sensory processing (Schroeder and Lakatos, 2009). This idea has received recent experimental support suggesting a prominent role of delta-theta oscillations in sensory selection (see below). However, the role of beta activity in such processes remains unclear.

While beta oscillations are classically related to motor processes, they are also involved in cognitive functions, and more specifically in tasks requiring endogenous, top-down control procedures (Engel and Fries, 2010). The results from Fujioka et al. (2012) in fact support the hypothesis that beta post-inhibitory rebound reflects a “predictive” modulation of auditory excitability and may result from the propagation of top-down predictions about forthcoming stimuli. The same authors previously showed a stronger increase of beta rebound with the omission of an expected sound (Fujioka et al., 2009). This is consistent with the hypothesis of a functional role of beta oscillation in the backward propagation of updated predictions when predictions are incorrect (Arnal et al., 2011). The origin of such descending signals is unknown, but recent evidence suggests that low-frequency oscillations may interact with higher (beta) ones to control sensory excitability during predictive timing.

### Role of low frequencies in predictive timing

Expecting a sensory event induces a phase-alignment of pre-stimulus low-frequency (delta-theta) activity (Lakatos et al., 2008). This mechanism permits the system to align cortical excitability with upcoming events and facilitates sensory processing. It accounts for anticipated behavioral responses to expected events and may also be at the origin of reduced early-evoked responses to predicted stimuli (Arnal and Giraud, 2012). While Fujioka et al. (2012) did not focus on low-frequency oscillations, recent evidence shows that during temporal expectations, beta-band power is coupled with delta-band phase-modulations in sensorimotor regions (Cravo et al., 2011; Saleh et al., 2011). This pre-stimulus alignment of low-frequency oscillations also relates to the contingent negative variation, an electrophysiological component implicated in predictive timing and presumably generated in the motor system. While there is currently no clear-cut experimental evidence for such a direct motor-to-sensory coupling, these results suggest that the motor system may play a key role in perception by “predictively” modulating sensory excitability via oscillatory coupling mechanisms.

## Efferent Signals from the Motor System Control Predictive Timing in Auditory Cortex

### Internal models in perception and action

The notion of internal models provides a unifying perspective about perception and action and involves the central idea that internal models predict (i) forthcoming sensory input or (ii) the sensory consequences of an action, respectively. In both cases, inferring sensory inputs allows minimizing their processing and classically leads to reduced early-evoked responses (Houde et al., 2002; Costa-Faidella et al., 2011). In the context of action, this process has been proposed to rely on the generation of efferent signals that propagate from the motor-to-sensory systems. While the notion of internal models assumes that the same computational strategies may apply to perception and action, the commonalities between perceptual and motor systems remain unclear.

### Implication of motor systems in perception

The results of Fujioka et al. (2012) show that even when attention is not directed toward the beat, the motor system processes the auditory regularity and therefore, presumably plays a role in the control of time-based expectations. This is also consistent with recent findings showing that motor systems can be recruited during speech perception (Wilson et al., 2004; Edwards et al., 2010), therefore supporting the idea that motor-based predictive mechanisms may facilitate the processing of quasi-periodic (and therefore temporally predictable) signals such as speech for instance (Arnal and Giraud, 2012). Other theoretical work suggests that the motor system can contribute in a modulatory way to perceptual processing. Active sensing theory, for instance, suggests that the motor system can be used to predictively modulate attention to facilitate sensory selection (Schroeder et al., 2010). Other work even suggests that the motor system internally simulates future events to anticipate their occurrence and facilitates their processing (Schubotz, 2007).

### Predictive timing using efferent signals

According to this idea, the simulation starts with the generation of an efferent signal that targets sensory systems and suppresses the sensory consequences of the predicted input. Note that such efferent signals (sometimes referred to as “efference copies” or “corollary discharges” in the literature, see Crapse and Sommer, 2008 for a review) have been evidenced even when no action is actually executed, as for instance during mental imagery (Tian and Poeppel, 2010). In the context of Fujioka et al.’s (2012) experiment, this suggests that in the absence of any synchronized movement – and even when attention is directed away from the auditory stream – the motor system actively simulates a movement synchronized with the beat and generates efferent signals at the tempo of the stimulation. As a consequence, “descending” neural signals that are usually used to suppress the sensory consequences of self-generated sounds could also be exploited to temporally predict the occurrence of a sound. That the authors observed a relation between the profile of beta rebound and the beat rate may reflect that slow and fast rhythms are tracked using larger and smaller simulated movements, respectively. Consistent with the notion that beta activity could be used in top-down processing (Engel and Fries, 2010; Wang, 2010; Arnal et al., 2011), *efferent signals may use a beta frequency channel* to predictively constrain neural processes in the auditory cortex. While beta oscillations may not be the unique mediator of such efferent signals and possibly interacts with lower-frequency bands, these findings suggest a new possible role of the motor system and its beta oscillations in perception.
